# Why technology matters as much as science in improving healthcare

**DOI:** 10.1186/1472-6947-12-103

**Published:** 2012-09-10

**Authors:** Robert J Szczerba, Marco D Huesch

**Affiliations:** 1 Lockheed Martin Corporation, Owego, NY 13827, USA; 2 USC Leonard D. Schaeffer Center for Health Policy and Economics, University of Southern California, Los Angeles, CA, 90089-7273, USA; 3 USC Sol Price School of Public Policy, University of Southern California, Los Angeles, CA 90089-7273, USA; 4 Health Sector Management Area, Duke Fuqua School of Business, Durham, NC 27708, USA

## Abstract

**Background:**

More than half a million new items of biomedical research are generated every year and added to Medline. How successful are we at applying this steady accumulation of scientific knowledge and so improving the practice of medicine in the USA?

**Discussion:**

The conventional wisdom is that the US healthcare system is plagued by serious cost, access, safety and quality weaknesses. A comprehensive solution must involve the better translation of an abundance of clinical research into improved clinical practice.

Yet the application of knowledge (i.e. technology) remains far less well funded and less visible than the generation, synthesis and accumulation of knowledge (i.e. science), and the two are only weakly integrated. Worse, technology is often seen merely as an adjunct to practice, e.g. electronic health records.

Several key changes are in order. A helpful first step lies in better understanding the distinction between science and technology, and their complementary strengths and limitations. The absolute level of funding for technology development must be increased as well as being more integrated with traditional science-based clinical research. In such a mission-oriented federal funding strategy, the ties between basic science research and applied research would be better emphasized and strengthened.

**Summary:**

It bears repeating that only by applying the wealth of existing and future scientific knowledge can healthcare delivery and patient care ever show significant improvement.

## Background

More than half a million new items of biomedical research are generated every year and added to Medline. How successful are we at *applying* this steady *accumulation* of scientific knowledge and so improving the practice of medicine? The conventional wisdom is that the US healthcare system is plagued by serious cost, [1] access, [2] safety, [3] fairness, [4] and quality [5] weaknesses. In combination, these many weaknesses yield a system which provides sub-optimal value [6,7].

A comprehensive solution must involve the more efficient translation of an abundance of clinical research into improved clinical practice [8,9]. Yet the application of knowledge (i.e. technology) still remains far less well funded and less visible than the generation, accumulation and synthesis of knowledge (i.e. science), [10] and the two are only weakly integrated despite attempts to spur translational research with large NIH Translation awards [11]. Worse, technology is often seen merely as an adjunct to practice, e.g. electronic health records. In this paper we discuss these issues and suggest potential solutions.

## Discussion

### Funding for translation of research

The Agency for Healthcare Research and Quality (AHRQ) is the lead federal agency sponsoring the application of health services knowledge, but has a budget of less than $400 million per year. Adding the various translational research budgets in the National Institutes of Health (NIH) comes to less than $1 billion per year, with a focus predominantly on therapeutics as opposed to health services and care delivery. Despite unprecedented support at NIH’s most senior levels for translational research,^11^ and a commitment of around $500 million per year for the Clinical and Translational Science Awards, [12,13] there is still no empirical evidence that clinical research has actually become more efficient [14]. Given a total of nearly $32 billion in funded research, [15] the amount and proportion spent implementing medical knowledge and improving care delivery may be too small.

Several key changes are in order. We argue that the absolute level of funding for technology development must be increased as well as being more integrated with traditional science-based clinical research. In such a more mission-oriented federal funding strategy, the ties between basic science research and applied research would be better emphasized and strengthened. A helpful first step lies in better understanding the distinction between science and technology, and their complementary strengths and limitations.

### The distinction between science and technology

*Science* is generally considered the search for and construction of theories about *cause*[16]. Science generates, synthesizes and accumulates knowledge in one narrow area; its models hold much else ‘fixed’. On the other hand, technology is the search for and production of theories about *new processes*. Technology is the application of existing knowledge; its models allow most everything to vary. Simply put, science is the ‘why’, while technology is the ‘how’ of improvement.

Advances in scientific knowledge in medicine are well-known and manifold and steady scientific progress will continue to lead to incremental improvements in accumulated knowledge. But only the appropriate and effective application of technology, through successful modification and application of existing knowledge, can realize the promised gains in cost, safety and efficacy.

In the health care domain, however, technology is best known as an enabler of process standardization and communication between providers [17]. The federal government’s Health Information Technology for Economic and Clinical Health (HITECH) Act reflects this important strength of technology, and rightly seeks to improve practice by significant technology investments.

### The full potential of technology

Yet electronic health records and process control do not capture the full potential of what technology can offer. For example, by integrating existing knowledge from behavioral science, organization science, engineering and clinical research and applying this in simulated environments, technology can improve and redesign existing care processes as well as engineer new ones.

More generally, technology allows researchers and stakeholders to overcome the inherently complex, interacting and dynamic nature of healthcare systems [18]. It is difficult to grasp all the linkages and interactions between humans, equipment, medical devices, care processes and biological systems. Faced with the need to improve such chaotic systems scientists simplify the problem and abstract the clinical setting. Clinical research routinely and deliberately seeks to hold many aspects of the problem fixed, and attempts to estimate the positive impact of making a small, isolated change in one component (e.g. the maximum hours worked by a provider).

This approach of holding many factors constant, and making small, incremental changes in a small number of factors is described as local optimization. However, such local optimization may hide the much larger impact of global optimization: making a larger number of coordinated changes to multiple components at once (e.g. the configuration of a medical instrument, the training of a provider *and* the mix of patients admitted to the system).

To view this conceptually, consider a system’s performance as being highly non-linearly impacted by two factors. Here non-linearity signifies that the response of a system to one factor changes both with the level of that factor and the level of the other factor, and that those responses vary in a complex way. One way to visualize this is by taking a three dimensional coordinate system. Plot the levels of the two factors in the two horizontal dimensions and let performance be measured in the vertical dimension.

The non-linearity of the relationships between the two factors and overall performance leads to a rugged ‘performance landscape’ with hills, ridges and valleys representing respectively better performance, knife-edge performance and worse performance for particular combinations of the two factors [19]. Local optimization can be represented as slow and steady progress up small ‘hills and ridges’, following the simple rule that ‘a little further is better’. Clearly, this type of optimization can lead to the system getting stuck at sub-optimal performance levels. Global optimization of would represent the skipping of small hills, the traversing of deep valleys, on the way to attaining the highest peak that represents optimal performance.

There is a trade-off between results and effort in contrasting these two different types of optimization. Local optimization is straightforward: for example, a simple regression analysis will suggest that a factor is significantly related to overall performance. However, global optimization often requires advanced simulation and modeling technology. Similar to other complex domains, these approaches are indispensable in a clinical context since the results of hypothesized changes to multiple system components cannot be predicted ahead of time and require *in vitro* experimentation.

### An illustrative example

Consider the differences between traditional research and applied research in, for example, improving the quality of intensive care for cardiovascular disease. A common clinical imperative is to measure and reduce variability and improve end outcomes through process standardization and improvement [20]. Classical use of static regression-based models, no matter how detailed, is unlikely to capture the complexity of cardiac surgery processes or the interactions between providers and hospitals [21]. Surveys and case studies may only isolate some of the key success factors that allow hospital cath labs to improve coronary intervention processes, [22] and not be able to model all the interactions. These methods are essentially all local optimization techniques, familiar and easy to use. However, as local optimization techniques, they are thus prone to missing potentially far more significant improvement opportunities.

On the other hand, a holistic simulation approach, using real-time data, could allow for safe experiential learning and experimentation, and thus significant improvements in the quality of such intensive care [23]. Coupled with multidisciplinary and trans disciplinary teams, this less familiar and less widely used approach would offer a more global optimization.

This trade-off between the ease of approach and potential benefits may also partly underpin the well-known phenomenon of the ‘flattening of the curve’ that represents US progress on health outcomes over time. Despite continual advances in accumulated knowledge, healthcare system performance often appears to have reached a plateau in the US [24]. This is likely due to the complex interplay between social determinants of health such as education, knowledge, income, and location [25]. To some degree, the ‘flattening’ of the value curve is also likely also due to the medical epidemics of diabetes, [26] obesity, [27] especially amongst the young. We speculate, however, that the flattening of the performance curve is partly also due to the delivery system’s failure to adequately apply existing clinical knowledge, or its application using local rather than global optimization techniques.

### Increasing the application of scientific knowledge

Clinical medicine is becoming increasingly comfortable with the use of technology to store patient data and guide the provision of care [28]. A similar technology-enabled shift towards deeper and more consistent application of existing scientific knowledge is necessary. While there is an emerging appreciation of the need for different, [29] more systems-based research, [30] most US federal funding for healthcare remains science-, and not technology-based [9].

To redress this, we believe, requires an unavoidable change in funding priorities and a substantial increase in the level of funding for technology development and the application of existing scientific knowledge. Equally important is how such scarce funding resources are allocated. Merely raising the proportion of NIH funding for applied research or increasing AHRQ’s limited budget will not suffice, especially if funding is generally limited to projects involving the adoption of commercial off the shelf based technologies such as electronic health records.

To truly reap the returns on science-based research, the increased funding for technology development must also be better integrated with science-based research. Adapting the mission orientation of the Department of Defense, basic clinical science research must also include technology transition plans. In this different paradigm, research conducted ‘upstream’ with the objective of increasing scientific knowledge must be held closer to account in terms of ‘downstream’ applications and ultimately delivering mission-critical performance.

While our focus in this article is on the US health system, it is noteworthy that other large healthcare systems have wrestled with similar concerns and have implemented similar recommendations as ours. For example, in the UK, the proportion of total funded research activity accounted for by basic biomedical research was most recently estimated as approximately two thirds of which the majority was laboratory-based biomedical research [31]. Another sixth of UK health research spending went to treatment development and evaluation, which includes applied and translational research.

In the Cooksey Report, Sir David Cooksey examined publicly funded healthcare research and sought to provide recommendations to optimize its potential to benefit patients, the National Health Servie and the wider healthcare economy, on behalf of the UK Treasury. This inquiry found that the UK risked failing to reap the full economic, health and social benefits of the large investments being made by taxpayers into health research [31].

In particular, the report diagnosed an absence of an overarching strategy to translate ideas from basic and clinical research into the development of new products and approaches to treatment of disease and illness. Similarly, another key gap noted was the absence of a strategy to implement those new products and approaches into clinical practice. Both gaps are ones we argue are still present in the US healthcare system.

To a more limited extent the Report’s recommendations on prioritizing projects in view of potential downstream significance are also in line with our recommendation to insist on an implementation plan for all basic research, tying them more closely to applied research [31]. Beyond this, the UK is seeking to implement cultural and organizational changes in the delivery system and in the research enterprise to better close the gaps between basic and applied science, and theory and practice [31].

To a large extent, the degree of non-market control of research and practice by centralized decision-makers is arguably much stronger in the UK’s essentially nationalized delivery system with centralized cost and comparative effectiveness functions. We are not convinced that changes to practice in the US will occur through organizational and cultural changes alone. We believe that demand-side consumer incentives and supply-side payment reform as well as institutional innovation in the markets for insurance and provision of care will be required [32].

## Summary

We remain convinced that system performance can improve and that any real reductions of the returns to investment or innovation are still far off. This performance deficit could, we argue, be addressed by implementing and applying some of those half a million items of scientific knowledge recently accumulated. This would be a good start to improving the practice of medicine in the US. These transformational improvements in the practice of medicine could have positive impacts in other countries around the world as well, as dissemination of applied knowledge and innovations improves [33].

The next step is making sure that the next million funded pieces of scientific research are also coupled to technology applications to the fullest extent possible. It bears repeating that only by applying the wealth of existing and future scientific knowledge can healthcare delivery and patient care ever improve.

## Competing interests

The authors declare that they have no competing interests.

## Authors’ contributions

Both authors are equally responsible for the paper. Both authors read and approved the final manuscript.

## Pre-publication history

The pre-publication history for this paper can be accessed here:

http://www.biomedcentral.com/1472-6947/12/103/prepub
